# The relationship between orexin levels and blood pressure changes in patients with narcolepsy

**DOI:** 10.1371/journal.pone.0185975

**Published:** 2017-10-12

**Authors:** Mariusz Sieminski, Kamil Chwojnicki, Tomi Sarkanen, Markku Partinen

**Affiliations:** 1 Department of Adults’ Neurology, Medical University of Gdansk, Gdansk, Poland; 2 Vitalmed Helsinki Sleep Clinic, Helsinki, Finland; Associazione OASI Maria SS, ITALY

## Abstract

**Study objective:**

Narcolepsy type 1 (NT1) is caused by a deficiency or absence of the neurotransmitter orexin. NT1 is also associated with a reduced nocturnal “dipping” of blood pressure (BP). The study objective was to analyze whether nocturnal BP values differed in patients depleted of orexin, versus those in whom production was preserved.

**Methods:**

We performed a retrospective analysis of the polysomnographic recordings, orexin levels, and BP values of patients with NT1. Data was collected from a total of 21 patients, divided into two groups as follows: those with a complete depletion of orexin (n = 11) (Group1), and those with a remaining, limited presence of orexin (n = 10) (Group 2).

**Results:**

The groups did not differ in terms of the clinical features of NT1 or sleep characteristics, with an exception of increased number of cataplexy episodes and increased percentage of sleep stage 2 in the Group 1. Daytime and nocturnal BP did not differ between the groups. Most patients, regardless of group, had a non-dipping blood pressure pattern, and no difference in dipping prevalence was observed between groups. The amplitude of the daytime to nighttime change in BP did not differ between the groups.

**Conclusions:**

Non-dipping BP patterns are frequent among patients with narcolepsy type 1, but we saw no evidence that they depended on whether orexin levels were above or below the assay detection threshold. Therefore, our results do not support the hypothesis that in patients with narcolepsy type 1 residual orexin levels play a role in the control of nocturnal BP dipping.

## Introduction

Narcolepsy (NC) is a chronic neurological disorder, clinically characterized by the presence of excessive daytime sleepiness, cataplexy, sleep paralysis, hypnagogic hallucinations, and disturbed nocturnal sleep. [1,2] These symptoms are caused by absent, or decreased levels of orexin (hypocretin)—a hypothalamic neuropeptide that helps control the circadian rhythms of sleep and wakefulness, as well as the processes of feeding and temperature regulation in the body.[3,4] As a result, patients with NC are unable to effectively control sleep-wake cycles. Orexin is produced by small population of hypothalamic neurons, with projections to numerous regions of the brain, that are responsible for transitions between specific states of sleep and wakefulness.

Additionally, the circadian rhythms of sleep and wakefulness are strongly related to changes in autonomic and cardiovascular functioning of the organism. One of the most prominent examples of this relationship is the nocturnal dipping of blood pressure (BP)—a significant lowering of BP values during the night.[5] The significance of this phenomenon has been described in many epidemiologic studies. Nocturnal decrease in BP to less than 10% of daytime values (so-called “non-dipping”) is a strong predictor of cardiovascular morbidity and mortality.[6–9] Even more important factor influencing the vascular risk is decreased value of mean BP during sleep compared to wake period as I was shown by Hermida et al. [10]While the neuronal and hormonal mechanisms underlying nocturnal dipping are not yet fully explained, they must, to some extent, overlap with the mechanisms responsible for transitioning between sleep stages.

Transitioning deeper into sleep, from stage 1 to the slow-wave stage, is accompanied by a consequent reduction in BP values, as well as an increase in the cardiovascular activity associated with cortical arousal during the REM period. Since orexin plays critical role in arousal and sleep regulation, these observations suggest that orexin may be involved in controlling nocturnal BP—a hypothesis for which a number of studies have provided support.[11,12] Intracerebral injections of orexin have been shown to increase BP values, whereas animals depleted of this neurotransmitter are characterized by lower BP values.[4,13–15] It must be also noted that in some studies orexin-depleted animals had blunted nocturnal dip of BP and higher values of BP during sleep (NREM and REM). [16–18] At present, three studies that focus on the interaction between nocturnal BP dipping and narcolepsy in humans have been performed. In two of them, nocturnal BP dipping was absent or blunted in patients with narcolepsy, compared to a control group of healthy subjects.[19,20] In the third study, non-dipping was shown to occur equally in a small group of narcoleptic and insomnia patients, suggesting that sleep disturbance, rather than an absence of orexin, diminishes BP dipping.[21]

The aim of this study was to further clarify this issue and determine if orexin has a clinically significant influence on the values of nocturnal BP. To accomplish this, we performed a retrospective analysis of the polysomnographic (PSG) recordings, orexin levels in the cerebrospinal fluid, and BP values of patients with NT1. We focused on the differences between daytime and nocturnal BP, and assessed whether nocturnal BP values differed in patients depleted of orexin versus those in whom production was preserved.

## Methods

The study protocol was accepted by the Independent Bioethical Committee for Scientific Research at the Medical University of Gdansk. Patients’ data was anonymized prior to analysis.

### Patients

Data was collected from patients of the VitalMed Helsinki Sleep Clinic who were diagnosed with NT1, underwent diagnostic multiple sleep latency test (MSLT), and who were HLA- DQB1*0602 positive. Of the 121 subjects in the clinic, 68 had the orexin levels in their cerebrospinal fluid measured. Of this population, we excluded patients due to the following criteria: below the age of 18 at the time of diagnostic workout and/or a diagnosis of cardiovascular diseases (e.g. hypertension, coronary heart disease). Of the remaining 44 subjects, 21 had full night PSG with beat-to-beat BP measurements available. PSG recordings with BP measurements were performed in all consecutive patients undergoing diagnostic procedures due to suspected narcolepsy, since the continuous BP measurement was available. Therefore there was no selection bias. Therefore, we included a total of 21 subjects in our study. All that patients were drug naïve at the time of PSG recordings. None of them was treated chronically due to cardiovascular diseases, including intake of antihypertensive drugs. Eleven subjects had orexin levels equal to 0 (Group 1), and 10 had orexin levels between 27 and 110 pg/ml(Group 2), with one subject with orexin level 127 pg/ml with typical clinical picture of NT1 with cataplectic episodes and pathological MSLT. Analysis of CSF orexin concentration was done at the Rinnekoti Research Centre (Orexin A RIA kit, Phoenix Pharmaceuticals, San Mateo, CA). Double measurements were always done in order to be sure about the level. If the levels varied we took the mean of the two values. The level 0 was used only if there were no hcrt in both measurement. It must be noted anyway that the accuracy of the assay is limited at concentrations of orexin below 30 pg/ml.

### Recordings and scoring

Patients underwent a single PSG study. All the recordings were performed with a SOMNOscreen plus PSG system (Somnomedics, Randersacker, Germany). Sleep recordings included four electroencephalographic (EEG) leads, two bilateral electro-oculogram leads (EOG), bilateral electromyographic leads (EMG) placed on the chin, and two surface EMG leads placed on the left and right anterior tibialis muscles, which recorded periodic limb movements (PLMs) during both sleep and wake. Respiration was recorded with a nasal cannula, through thoracic and abdominal strains, and by finger oxymetry. Electrocardiogram (ECG) was recorded with single precardial lead. The PSG included non-invasive, beat-to-beat BP measurements that were performed in conjunction with pulse transit time (PTT). [22] The PSG recordings were scored according to the American Academy of Sleep Medicine guidelines.[23]

### Cardiovascular assessment

BP measurements were based upon pulse transit time (PTT) measurements. There was a finger photoplethysmograph (also recording oxygen saturation) attached to a patient’s finger. Beat-to-beat PTT is measured as the time between R-wave on ECG and the moment of detection of corresponding pulse wave by the finger photoplethysmograph. [24] A mathematical model describing relation between PTT and BP values [22] and results of traditional measurement of BP (used for calibration of the measuring system) allowed to calculate the values of BP. This method was recently validated against conventional measurements with cuff sphygmomanometer. [24] The mean difference between cuff-based BP measurement and PTT-beased measurement was in this study 0.44±6.1 mmHg for systolic BP and 0.33±3.4 for diastolic BP. It was also validated against oscillometric method during application of CPAP [25].

For the calibration of the system an auscultatory BP measurement with sphygmomanometer was performed on the nondominant arm before the start of the recording. The results of the measurement were noted. The auscultatory BP measurement was repeated at the end of the recording.

For the assessment of the cardiovascular system, the mean systolic and diastolic BP (SBP and DBP, respectively) were calculated during the day (time from the start of the recording until the “lights-off” moment, and from the “lights-on” moment until the end of the recording) and during the night. It was 30 minutes period, with patients staying awake in bed, before switching off the lights. Additionally, the amount by which the mean values of both SBP and DBP were reduced or “dipped” during the night, as compared to the day, and the percentage by which they dipped, were calculated. Patients whose mean blood pressure (SBP or DBP) decreased during the night by less than 10% were considered “non-dippers”. Similar analysis was performed for wake and sleep periods.

### Statistical analysis

Categorical variables were compared between the groups with Fisher’s exact test. Continuous variables were compared between the groups with the Mann-Whitney-Wilcoxon test. *P* values lower than 0.05 were considered statistically significant. To detect any other factors possibly contributing to values of nocturnal dip of systolic and diastolic blood pressure Pearson correlation coefficients (r) were calculated for variables that may impact BP. The analysis was performed for the whole study population (n = 21). Pearson correlation coefficient was calculated for each of selected variables and the value of percentage dip in systolic and diastolic BP.

Calculations were performed using SAS statistical software.

## Results

The clinical features of the patients are shown in Table 1. Briefly, there were 11 patients in the Group 1 and 10 patients in the Group 2. The groups did not differ in terms of age or gender distribution, and all patients were positive for haplotype HLA-DQB1*0602. Additionally, the groups did not differ in their age of onset of NT1, disease duration, or respective clinical features of NT1, with the exception of a higher number of cataplexy episodes per week in the Group 1 (Table 1).

**Table 1:** Clinical features of patients from both groups.

	Group 1 (n = 11)	Group 2 (n = 10)	p
**Gender (M/F)**	2/9	3/7	0,63
**Age (years; mean±SD)**	28.1±14.7	29.7±10.9	0.39
**BMI (kg/m**^**2**^**; mean±SD)**	25.3±4.6	28.7±6.2	0.08
**Years from NT1 diagnosis (years; mean±SD)**	6.0±5,3	9.1±9.0	0.17
**Number of cataplexy episodes per week (mean±SD)**	18.4±23.7	5.3±5.5	0.05
**Sleep Latency on MSLT (minutes, mean±SD)**	5.4±4.7	6.4±5.3	0.32
**Number of SOREMPs (mean±SD)**	2.4±1.4	1.7±1.1	0.12
**Presence of sleep paralysis**	5/11	2/10	0.36
**Presence of nightmares**	10/11	6/10	0.15
**Orexin Level (pg/mL; mean±SD)**	0.0±0.0	62.3±32.1	<0.0001
**ESS at the moment of PSG (mean±SD)**	16.8±3.0	15.1±3.1	0.2

SD—standard deviation; NS—not significant; NT1 —narcolepsy type 1; MSLT—multiple sleep latency test; SOREMPs—sleep onset REM periods; ESS—Epworth sleepiness scale;

The sleep characteristics of the groups are shown in Table 2. These characteristics did not differ between the groups, with the exception of a higher percentage of total sleep time that was spent in sleep stage 2 in the Group 1 (Table 2).

**Table 2:** Sleep characteristics of both groups.

	Group 1	Group 2	p
**TST (minutes; mean ± SD)**	424.2± 55.2	461.1±72.4	0.1
**SE (%) (mean ± SD)**	84.6±10.0	89.0±9.9	0.2
**Sleep Latency (minutes; mean ± SD)**	13.0±16.1	13.6±21.2	0.5
**Sleep stage 1 (%) (mean ± SD)**	18.1±12.1	14.4±4.7	0.2
**Sleep stage 2 (%) (mean ± SD)**	44.6±8.7	35.8±9.9	0.02
**SWS (%) (mean ± SD)**	19.1±8.8	23.6±10.4	0.1
**REM (%) (mean ± SD)**	36.6±54.3	26.1±3.4	0.3
**AHI (mean ± SD)**	2.4±2.0	6.4±14.4	0.2
**PLMS-I (mean ± SD)**	7.3±5.8	6.5±6.7	0.4
**PLMSA-I (mean ± SD)**	1.0±1.6	1.9±3.0	0.2
**Sleep stage change index (mean ± SD)**	15.2±7.1	13.4±5.3	0.3
**Arousal Index (mean ± SD)**	16.6±8.3	17.7±8.2	0.4
**Wake index (mean ± SD)**	3.7±1.8	3.3±2.0	0.3
**WASO (mean ± SD)**	67.4±44.3	57.4±33.1	0.3

TST—total sleep time; SE—sleep efficiency; SWS—slow wave sleep; AHI—apnea-hypopnea index; PLMS-I—periodic limb movements in sleep index; PLMSA-I—periodic limb movements with arousal index; WASO—time of wake after sleep onset; SD—standard deviation.

Similarly, the groups did not differ in terms of daytime and nocturnal SBP and DBP, as shown in Table 3. Both groups exhibited a nocturnal dip in BP values; however, dip values rarely reached 10% of the daytime BP. With respect to SBP, nine patients in the Group 1 were classified as non-dippers, and six in the Group 2 (non-significant difference, P>0.05; Table 3). With respect to DBP, eight patients in Group 1 were considered non-dippers, and seven in Group 2 (non-significant difference, P>0.05; Table 3). One patient in the Group 1, and three patients in the Group 2 exhibited a nocturnal dip in both SBP and DBP values of greater than 10%. There was no statistically significant difference in the value of nocturnal dip (both in mmHg and in %) between the groups (Table 3).

**Table 3:** Blood pressure measurements and nocturnal dipping in both groups.

	Group 1	Group 2	p
**Manually recorded initial SBP (mmHg; mean±SD)**	124.5± 11.9	127.0±9.8	0.3
**Manually recorded final SBP (mmHg; mean±SD)**	114.5±9.3	115.5±9.8	0.4
**Manually recorded initial DBP (mmHg; mean±SD)**	73.6±7.4	75.1±13.3	0.4
**Manually recorded final DBP (mmHg; mean±SD)**	70±10.9	71.0±14.9	0.4
**Daytime SBP (mmHg; mean±SD)**	122.0±13.4	127.3±11.3	0.17
**Nocturnal SBP (mmHg; mean±SD)**	115.5±13.5	116.6±12.9	0.4
**Dip of SBP (mmHg; mean±SD)**	6.4±5.7	10.7±7.9	0.14
**Dip of SBP (%; mean±SD)**	5.2±4.5	8.4±6.2	0.14
**Daytime DBP (mmHg; mean±SD)**	73.0±7.6	73.3±13.6	0.48
**Nocturnal DBP (mmHg; mean±SD)**	69.6±9.0	70.3±15.3	0.45
**Dip of DBP (mmHg; mean±SD)**	3.4±4.6	3.0±5.3	0.89
**Dip of DBP (%; mean±SD)**	4.9±6.5	4.4±8.0	0.89
**Non-REM SBP (mmHg; mean±SD)**	109.9±13.4	113.7±12.2	0.25
**REM SBP (mmHg; mean±SD)**	117.2±14.3	121.2±13.0	0.25
**Non-REM DBP (mmHg; mean±SD)**	64.1±7.1	64.2±13.4	0.49
**REM-DBP (mmHg; mean±SD)**	66.9±7.4	67.0±14.0	0.45

SBP—systolic blood pressure; DBP—diastolic blood pressure; SD—standard deviation.

There was also no difference between the groups in terms of values of blood pressure during wake and sleep periods. Lack of wake/sleep dipping was also noticeable in both groups but again without any significant difference between them. Results of comparisons of blood pressure during wake and sleep periods are shown in Table 4.

**Table 4:** Blood pressure values during wake and sleep periods.

	Group 1	Group 2	P
**Wake-period SBP (mmHg; mean±SD)**	120.8±14.7	125.0±13.4	0.2
**Sleep-period SBP (mmHg; mean±SD)**	115.5±13.5	116.4±13.0	0,4
**Wake/Sleep Dip of SBP(mmHg; mean±SD)**	5.3±6.0	8.6±8.3	0.1
**Wake/Sleep Dip of SBP (%; mean±SD)**	4.2±4.7	6.7±6.3	0.2
**Wake-period DBP(mmHg; mean±SD)**	72.0±7.9	72.1±15.0	0.5
**Sleep-period DBP (mmHg; mean±SD)**	69.6±10.0	70.2±15.3	0.5
**Wake/Sleep Dip of DBP(mmHg; mean±SD)**	2.4±4.0	1.9±5.0	0.4
**Wake/Sleep Dip of DBP (%; mean±SD)**	3.5±5.7	2.6±7.4	0.4

As there was one patient in group 2 with highly increased value of AHI, which might have some impact on the values of BP and its dipping, we have performed a parallel analysis, with exclusion of that subject. The groups still did not differ significantly in terms of values of nocturnal dipping (systolic dip (%): 4.2±4.7 vs. 6.4±6.6, p = 0.2; diastolic dip (%): 3.5±5.7 vs. 2.3±7.7, p = 0.3)

Both groups did not differ in terms of number of patients with elevated mean values of blood pressure during daytime and nighttime. The normative values were taken accordingly to European Society of Hypertension guidelines: normal mean values of systolic blood pressure: <135 mmHg (daytime) and <120 mmHg (nighttime); normal mean values of diastolic blood pressure: <85 mmHg (daytime) and < 70mmHg (nighttime).[26] The numbers of patients with increased values of mean BP are shown in Table 5.

**Table 5:** Numbers of patients with increased mean values of blood pressure.

	Group 1	Group 2	P
**Number of patients with increased daytime SBP**	2	2	1.0
**Number of patients with increased nighttime SBP**	3	3	1.0
**Number of patients with increased daytime DBP**	0	2	0.2
**Number of patients with increased nighttime DBP**	7	5	0.7

Values of Pearson correlation coefficients for correlation between variables that potentially may impact the values of the nocturnal dip of blood pressure are presented in Table 6. The only significant (negative) correlation was found between age of the patients and values of dip of systolic and diastolic BP (P<0.05).

**Table 6:** Pearson correlation coefficients for correlations between patients’ characteristics and percentage of dip of systolic and diastolic blood pressure.

	% of nocturnal dip of systolic blood pressure	% of nocturnal dip of diastolic blood pressure
**Age**	- 0.56	- 0.57
**BMI**	0.05	- 0.18
**Total sleep time**	0.17	0.10
**Sleep efficiency**	0.02	0.11
**Wake Index**	-0.09	-0.26
**WASO**	-0.05	-0.28
**AHI**	0.12	-0.05
**PLMS-I**	0.26	0.36
**Orexin concentration**	0.1	-0.21

We also analyzed wake/sleep differences in R-R intervals in the ECG recordings. R-R intervals were longer during sleep period in both groups. Increase of R-R interval was smaller in Group 1. None of those differences reached statistical significance. Results are shown in Table 7.

**Table 7:** Wake/Sleep differences in R-R intervals.

	Group 1	Group 2	P
**Wake period R-R interval (ms; mean±SD)**	844.8±106.4	801.0±68.8	0.14
**Sleep period R-R interval (ms; mean±SD)**	959.0±149.3	945.6±105.1	0.41
**Sleep/Wake R-R interval difference (ms; mean±SD)**	114.2±199.2	144.5±104.1	0.34
**Sleep/Wake R-R interval Difference (%; mean±SD)**	15.6±24.8	18.5±14.2	0.37

## Discussion

To the best of our knowledge, this is the first NT1 study to compare nocturnal BP between patients with the concentration of orexin above or below the detection threshold. We report two primary findings: 1) patients with narcolepsy have a decreased or absent wake-sleep reduction of their blood pressure, and 2) the nocturnal reduction of BP in these patients was not related to the presence of residual orexin in the cerebrospinal fluid.

Of the 21 patients in our study, only four subjects exhibited a nocturnal decrease in their SBP and DBP that was larger than 10% of their respective daytime values. While we did observe a noticeable drop in the BP values of patients in both groups, on average, they did not reach the threshold level of 10%. We also observed a similar pattern while analyzing difference between wake and sleep periods. There was no difference between the groups in this analysis as well. Those findings are in agreement with previously published studies performed on human subjects. In one, Grimaldi and colleagues compared 10 patients with narcolepsy to 12 healthy controls, and found a non-dipping pattern of BP in the narcolepsy group.[20] Similarly, Dauvilliers and colleagues, in a study in which 50 patients with narcolepsy were compared with 42 healthy controls, reported that the prevalence of non-dippers was increased among patients with narcolepsy.[19] A non-dipping pattern was also found by Donadio et el.[27] Furthermore, patients with narcolepsy have even been shown to have decreased dipping compared with other sleep disorders, such as insomnia.[21] However, when patients with NT1 were compared with patients with insomnia who did not differ significantly in terms of sleep characteristics, no difference in nocturnal dipping was found (non-dipping was equally prevalent in both groups).[21] The reduction of systolic BP was higher in the Group 2 but the difference did not reach the statistical significance—this result may be a consequence of small numbers of patients included in the study.

It must be remembered that we have analyzed only 30 minutes of daytime activity of the patients, before Lights Off moment. Taking into consideration 24-hour recording of BP could influence the final results. Nevertheless, our finding, as it was underlined above, are in agreement with studies using 24-hour BP recordings. [19,20]

We analyzed also wake/sleep change of ECG recording, comparing change between wake and sleep periods in R-R intervals. We have found a sleep–related prolongation of R-R intervals in both groups. The difference was smaller in orexin-depleted subjects than in patients with detectable levels of orexin, although the difference did not reach statistical significance. This results remains in agreement with previously published studies pointing at deranged cardiac control in NT1 patients. [20,27–29]

Finally, our results confirm similar observations made in animal studies; Bastianini and colleagues measured an increase in BP during sleep and a blunted nocturnal BP dip in hypocretin-deficient mice, compared to wild-type animals.[16] Taken together with the literature, our findings indicate that patients with NT1 exhibit a diminished nocturnal BP dip; however, the mechanism underlying this effect is not yet clear.

One possible explanation could be a disordered sleep architecture. In support of this, a study by Grimaldi and colleagues found that patients with NT1 were characterized by increased sleep fragmentation, arousals, and PLMs compared to controls—with the number of PLMs being significantly associated with reduced dipping.[20] Additionally, Dauvilliers and colleagues observed a significantly higher apnea-hypopnea index, and percentage of sleep spent in the REM stage, in the NC population. Moreover, they reported a negative correlation between nocturnal dipping and REM sleep (both the sleep percentage and sleep onset periods).[19] In a study by Baker and colleagues, a significantly higher BP in narcoleptic patients was reported, compared to subjects with idiopathic hypersomnia. Narcoleptic patients had also more apneic/hypopneic episodes and PLMS.[30]

Another possibility is that orexin deficiency influences the control mechanisms of the cardiovascular system, leading to a disordered nocturnal BP pattern. In this study, while all included patients were diagnosed with NT1, they differed in their respective orexin concentrations (orexin concentration below the detection threshold versus a decreased but detectable level of orexin). Importantly, we found that the amount of BP dipping and the prevalence of non-dippers, did not differ between groups. If orexin is responsible for the circadian patterns determining BP, then differences in its concentration should lead to differences in nocturnal BP changes. While it is possible that the clinical features of narcolepsy and/or sleep characteristics could also contribute, the groups in our study did not differ in either of those respects (except for a small difference in the percentage of sleep in stage 2). Instead, the primary difference between groups was the concentration of orexin in the cerebrospinal fluid. Therefore, our results indicate that orexin has a limited role in the control of nocturnal BP.

There is a third possibility—a threshold effect of orexin. It is possible that there is some (individually different) level of orexin above which the neurotransmitter may influence the cardiovascular system. As all the patients participating in our study had very low or undetectable level of orexin it is possible that the concentration of the neurotransmitter was too low in both groups to produce visible impact on values of BP or heart rate.

It is important to note, however, that our conclusion is at odds with studies performed in animal models. In one, Kayaba found that orexin knockout mice had lower values of BP compared to wild-type animals.[31] Similarly, Zhang and colleagues found that orexin neuron-ablated transgenic mice had lower BP values compared to wild-type controls.[32] Additionally, numerous studies using intracerebral injections of orexin have consistently reached the same conclusion: that orexin is a BP-increasing substance and, therefore, a deficit should decrease BP values.[33, 34] However, a study performed by Bastianini and colleagues found increased BP values and a blunted nocturnal BP dip in orexin-deficient mice.[16] Blunted sleep-related decreases in systolic or mean blood pressure were found in another animal studies [17,18]. Those results suggest that deficiency of orexin leads to reduction of sleep-related dipping of blood pressure but the exact mechanism of this relation was not found so far. On the other hand, evidence that sleep-related variability of BP (changes of BP values within one sleep state) is not orexin-dependent found by Silvani in patients with narcolepsy [35] may be to some extent interpreted as supportive for our results.

We have performed an analysis of correlations between various factors, such as age, BMI, sleep characteristics, orexin level and the values of the blood pressure dip. The only significant correlation was with age, which does not influence our conclusions, as both groups did not differ in terms of age.

There are two methodological limitations of our study. First, PTT-based BP recording during PSG still is not validated against traditional cuff-based method. Cited papers focusing on the validation of PTT-based BP measurements (24, 25) described a situation different from typical PSG recording. Moreover, according to Schmalgemeier et al. (25) the measurement can be biased by 4.1 mmHg for SBP and by 2.3 mmHg for DBP. This potential bias must be remembered while looking at wake/sleep differences in values of BP. On the other side, PTT-based measurements give the precision of beat-to-beat BP recording and allow avoidance of disturbing sleep with noise of the filling the cuff.

Second, we have compared BP values from 30 minutes of restful awakening and from the nighttime. Although this allows to observe wake/sleep difference in BP values, it must be remembered that to classify a patient as a “dipper” or “non-dipper” full 24-hour recording must be analyzed. An approach similar to ours (use of PTT and data collected from PSG recording) was recently used in a study focusing on relation between sleep apnea and BP in children.[36]

An a-priori power analysis was not performed which is a limitation of the study. This problem was discussed before the study and taking into consideration a small sample of available subjects decision of performing the study was taken. Another limitation of our study was the small number of participants, which is a result of the low prevalence of NT1, as well as of the criteria required for inclusion in the study. However, the rigoristic criteria used in this study, as well as the vigilant attention paid to the diagnostic procedures, allowed us to compare two NT1 patient groups that differed only in their orexin concentration. Another weakness of our study is lack of control group of healthy subjects with orexin level measured. It must be noted that reduction of nocturnal dipping in comparison to healthy subjects was already shown in previously published papers. [19,20] The only laboratory tests analyzed in the study were the ones related to NT1. Such factors as kidney functions or thyroid functions that may influence values of BP were not analyzed in this project. Another laboratory limitation of our study was the sensitivity of the orexin RIA assay used. Its accuracy is limited at concentrations below 30 pg/ml. There is therefore a possibility that patients could be misclassified between the groups although we undertook the procedures (double measurement for each sample) to minimize this risk.

In conclusion, our study showed that non-dipping BP patterns are frequent among patients with narcolepsy type 1 with detectable orexin level and in patients with orexin level below the detection threshold. We saw no evidence that non-dipping BP patterns depend on whether orexin levels were above or below the assay detection threshold. Therefore our results do not support the hypothesis that in patients with narcolepsy type 1 residual orexin levels play a role in the control of nocturnal BP dipping.

Since a lack of nocturnal dipping is related to an increased risk for organ damage, patients with narcolepsy should regularly undergo medical assessments that include 24-hour ambulatory BP measurements, echocardiography, and tests of kidney function.
